# Insights into the underlying mechanisms and clinical management of microscopic colitis in relation to other gastrointestinal disorders

**DOI:** 10.1093/gastro/goac011

**Published:** 2022-04-07

**Authors:** Yuanbin Liu, Mingkai Chen

**Affiliations:** Department of Gastroenterology, Renmin Hospital of Wuhan University, Wuhan, Hubei, P. R. China

**Keywords:** microscopic colitis, inflammatory bowel disease, celiac disease, irritable bowel syndrome, management

## Abstract

Microscopic colitis (MC) is a chronic inflammatory disease of the large intestine and as a relatively late recognized condition, its relationship with other disorders of the gastrointestinal tract is gradually being understood and investigated. As a multifactorial disease, MC interacts with inflammatory bowel disease, celiac disease, and irritable bowel syndrome through genetic overlap, immunological factors, and gut microflora. The risk of colorectal cancer was significantly lower in MC, gastrointestinal infections increased the risk of developing MC, and there was an inverse association between *Helicobacter pylori* infection and MC. A variety of associations are found between MC and other gastrointestinal disorders, where aspects such as genetic effects, resemblance of immunological profiles, and intestinal microecology are potential mechanisms behind the relationships. Clinicians should be aware of these connections to achieve a better understanding and management of MC.

## Introduction

Microscopic colitis (MC) is a chronic inflammatory disease of the large intestine that primarily affects the elderly and has a female predominance [1]. Persistent watery diarrhea is indicated as the main manifestation and the disease can be recurrent in course [2, 3]. MC is characterized by a nearly normal endoscopic appearance with microscopic abnormalities identified on histology—a feature that distinguishes it from classical inflammatory bowel disease (IBD) [4]. Histologically, it comprises two main types: one with an increase in lymphocytes in the sub-epithelial layer of the colon (≥20 lymphocytes/100 colonic epithelial cells) without a thickened collagen lamina termed lymphocytic colitis (LC), and the other type, collagenous colitis (CC), which is presented as lymphocytosis with a collagen band of >10 μm.

Since MC was first described in 1982, the incidence and prevalence have been increasing overall, with recent epidemiological studies showing that it has expanded many times compared with the situation in around 2000 [5–8]. MC constitutes ∼10%–15% of the population suffering from chronic diarrhea and has emerged as the leading cause of diarrhea in the elderly [9]. Aberrant response of the immune system to intestinal antigens is the key pathogenic mechanism of MC and a host of immune-related diseases are associated with it, such as type 1 diabetes, autoimmune thyroid disease, and other autoimmune disorders [10]. In the context of the digestive system, MC is also commonly associated with immune disorders such as celiac disease and IBD. Furthermore, MC is predisposed to developing in individuals with susceptibility genes that are shared with those implicated in IBD [4]. Several intestinal infections are followed by an increased risk of acquiring MC, putting this condition in contact with a couple of specific infections [11–13]. The overlap between irritable bowel syndrome (IBS) and MC in terms of symptoms and diagnostic methods also renders the two conditions confusing [14]. This makes for a cross-linked relationship between MC and these gastrointestinal disorders. Behind these disease relationships lies the clinical aspect of management, and the interaction between MC and them makes it more complex and comprehensive in terms of management.

Here, we aim to clarify the interfaces between MC and other digestive disorders, illustrate the mechanisms involved, and explore the impact on disease management. With the understanding of these aspects, we hope to offer a better insight into MC as a disease group and provide clinical perspectives on the precise location of MC in gastroenterology.

## Method

The electronic databases PubMed and Embase were retrieved manually to obtain relevant literature. The reference lists in the majority of the included literature were also checked internally to search for matches. Only publications in the English language were included. There was no restriction on the year of publication for the documents. The databases were queried using a combination of MeSH terms and entry terms, including “microscopic colitis,” “lymphocytic colitis,” “collagenous colitis,” “colitis,” “inflammatory bowel disease,” “irritable bowel syndrome,” “celiac disease,” “cancer,” “colon,” and “treatment.” All included publications were critically reviewed. Endnote X9 software was used for literature management.

## Possible proposed pathogenic mechanisms for MC

### An overview of the pathogenesis of MC

A recent systematic review provided a detailed summary of the possible pathogenic mechanisms involved in MC [10]. Although the etiology of MC remains unclear, an understanding of its plausible pathogenesis may allow a rational explanation of the link between MC and other digestive tract diseases. Therefore, a thorough overview of the proposed possible etiopathogenic mechanisms of MC is fundamental and crucial to facilitate understanding its interaction with related diseases. The pathogenesis of MC is summarized in Table 1.

**Table 1.** Proposed possible pathogenic mechanisms of MC

Proposed pathogenesis of MC	Altered components	Specific changes	Influencing factor	Mechanism description	Comments	References
Environmental factors	Smoking and alcohol consumption	Smoking: alteration of the gut microbiome, and inflammation induction; alcohol consumption: integrity of the intestinal epithelial barrier; endotoxin-producing bacteria	NA	Smoking: epithelial barrier dysfunction and intestinal inflammation; alcohol consumption: increasing the trans-epithelial and paracellular passage of luminal antigens, dysbiosis, and intestinal bacterial overgrowth	Smoking: a pooled OR of 2.99 for current smokers and 1.63 for former smokers compared with never smokers; alcohol consumption: aHRs of MC were 1.20 for consumers of 0.1–4.9 g/day of alcohol, 1.90 for consumers of 5–14.9 g/day, and 2.31 for consumers of ≥15 g/day	[15, 16]
Medication	PPIs, NSAIDs, statins, and SSRIs	Intraluminal environment and bacterial flora; bowel integrity and colonic permeability	NA	PPIs: acid suppression-related colonic dysbiosis and affected immune reaction; NSAIDs: intestinal damage and increased bile salt cytotoxicity; statins: unknown; SSRIs: aggravation of colitis symptoms by interference with the gastrointestinal motility and secretion	Poor understanding of the pathogenic mechanisms	[17–20]
Infectious agents	*Clostridioides difficile*, norovirus, *Escherichia* species, *Campylobacter concisus*, and H*. pylori*	Enteric flora; intestinal microenvironment	NA	Further dysbiosis of the enteric flora; intestinal epithelial sodium channel dysfunction and claudin-8-dependent gut barrier dysfunction; alteration of the intestinal microenvironment activates the immune pathway	*H. pylori* may reduce the risk of MC and the “hygiene hypothesis” may be the relevant mechanism.	[11, 12, 22–26]
Autoimmune disorders	HLA haplotypes; autoantibodies; hypersensitivity response	Concomitant autoimmune conditions and shared HLA genotype; serum antinuclear antibodies, IgM, antigliadin IgA, anti-endomysial, ASCA; drug or food allergy	Some drugs	Underlying autoimmune diseases affecting the gut or cross-reactivity of antigens due to increased intestinal permeability	No direct evidence	[10]
Genetic factors	Genetic predisposition	HLA region and the extended haplotype 8.1	NA	HLA-DQ2 as a shared genetic predisposition to celiac disease; haplotype 8.1 in association with collagenous colitis	HLA regions play a role in MC	[10]

aHR, adjusted hazard ratio; ASCA, anti-*Saccharomyces cerevisiae* antibody; HLA, human leukocyte antigen; *H. pylori*, *Helicobacter pylori*; MC, microscopic colitis; NA, not available; NSAIDs, non-steroidal anti-inflammatory drugs; OR, odds ratio; PPIs, proton-pump inhibitors; SSRIs, selective serotonin reuptake inhibitors.

### Risk factors associated with MC

Also, environmental factors such as smoking [15] and alcohol consumption [16], certain medications such as non-steroidal anti-inflammatory drugs (NSAIDs) [17], statins [18], selective serotonin reuptake inhibitors (SSRIs) [19], and proton-pump inhibitors (PPIs) [17, 20] have been shown to be associated with an increased risk of MC. These risk factors may therefore act as a contributing component in the pathogenesis of MC (Table 1).

### The possible role of gastrointestinal infections

Intestinal microflora dysbiosis in MC is one of the possible pathogenic mechanisms and therefore infections of the gastrointestinal tract, especially certain specific bacterial infections, could potentially be associated with the risk of MC. A previous systematic review found that gastrointestinal infections were involved in the risk of IBD, with several specific infections being associated with an increased risk of IBD and *Helicobacter pylori* infection reducing the risk of IBD [21]. There is supposedly also a risk profile for MC as a possible attenuated form of IBD associated with gastrointestinal infections.

In a nationwide case–control study, gastrointestinal infections were significantly associated with an increased risk of MC, with an adjusted odds ratio (OR) of 2.63 (95% confidence interval [CI], 2.42–2.85) [11]. Several specific infections, *Clostridium difficile*, norovirus, and *Escherichia* species, increased the odds of developing MC, whereas no association was found for *Salmonella* species. The increased risk due to gastrointestinal infections was higher in CC than in LC. Another cohort study demonstrated a significantly elevated risk of MC following *Campylobacter concisus* infection [12]. Several case series have also reported new-onset MC following recurrent *C. difficile* infection [22–24]. An inverse association was found between *H. pylori* infection and MC, similar to that in IBD [25, 26]. Differences in the prevalence of *H. pylori* in distinct regions may provide an explanation for the differing ethnic distribution of patients with MC [25].

Infection of the gastrointestinal tract leads to further dysbiosis of the enteric flora in patients with MC, initiating associated immune pathways and thus increasing the risk of MC. Specific infections such as *C. difficile* infection and *C. concisus* infection have a higher risk of developing MC, which seems to indicate that these bacteria have a more sustained pro-inflammatory effect [11, 12]. *Campylobacterconcisus* has also been found to be associated with intestinal epithelial sodium channel dysfunction and claudin-8-dependent gut barrier dysfunction—a dysregulation that leads to a translocation of the intestinal flora, making it even further dysregulated [27]. Alternatively, gastrointestinal infections may have activated immune pathways in MC by altering the intestinal microenvironment, since CC, a subtype in which more immune mechanisms are involved [28], is more strongly implicated in infections of the gastrointestinal tract.

Apart from epidemiological evidence, the protective effect of *H. pylori* infection on MC remains largely unknown at present. However, as a similar inverse association has been found in IBD, it is possible to speculate that the mechanisms involved may be consistent. In mouse models of experimental colitis, *H. pylori* exposure exhibits a blocking or mitigating effect on colitis [29]. In this context, the NLRP3 inflammasome and interferon-18 (IL-18) are involved in the protective mechanism of *H. pylori*. Meanwhile, helper T-cell (Th), Th17/Th1-related cytokines were found to be downregulated while cytokines secreted by Th2 were upregulated [30–32]. This suppression of pro-inflammatory cytokines is likely to be implicated in the protective mechanism [33]. This evidence suggests that *H. pylori* may reduce the inflammation of IBD through immunomodulatory effects. Interestingly, not all strains of *H. pylori* exhibit this effect. The specific component of *H. pylori*, CagA, may be an integral component of the protective mechanism. In patients with IBD who were seronegative for CagA, no significant protective effect was demonstrated [34].

Another plausible cause of this inverse relationship may be the “hygiene hypothesis.” This hypothesis was originally proposed by Strachan [35], who found that early sibling infections were associated with a decrease in future autoimmune diseases or allergies. Based on this hypothesis, some of the infectious agents that grow with us may be able to prevent the development of a range of immune-related diseases [36]. The clearance of *H. pylori* has been shown to lead to a disturbance of the intestinal flora of the colon [37]. A study showing the therapeutic effect of *Schistosoma mansoni* and Ancylostoma caninum soluble proteins on experimental colitis sidesteps this hypothesis [38]. Thus, the inverse association observed between *H. pylori* infection and MC may be attributed to that *H. pylori* acts as a surrogate marker of a commensal flora that reduces the occurrence of MC. However, no studies on MC are currently available and further validation of this hypothesis is needed in the future (Table 1).

## Similarities and differences in the genetic susceptibility and immunology of LC and CC

Although CC and LC are covered under the umbrella term MC and are similar in many aspects such as clinical presentation and prognosis, there are still some essential differences and hence the two diseases should be perceived as separate entities. They are distinguished by histological findings in pathological biopsies, and otherwise have marked distinctions at the level of immunology and susceptibility genes [10, 28, 39–43]. These molecular and cellular aspects may have contributed to their dissimilar relationship with other gastrointestinal diseases. Understanding these distinctions is consequently mandatory to appreciate the differences that may arise between CC and LC in this interaction. The similarities and differences in immunology and genetic susceptibility between them are synthesized in Table 2.

**Table 2.** Similarities and differences between CC and LC in terms of immunological profile and genetic susceptibility

Subtypes of MC	Immunological profile						Genetic predisposition	References
	Chemokine and receptor	Cytokine	Prostaglandin	Growth factor	T-lymphocyte	Others		
CC and LC	↑: CXCL8, CXCL9, CXCL10, CXCL11, CCL2, CCL3, CCL20, CX3CL1, CX3CL2, CXCR1, CXCR2	↑: TNF-α mRNA, IFN-γ mRNA, IL-17-A mRNA, IL-10, IL-21, IL-23	↑: COX-2	–	↑: Th1/Tc1, Th17/Tc17, Ki67+ T, CD45RO+ T, FoxP3+ Treg	↓: TRECs	–	[10, 39–42]
CC	↑: CXCR3, CX3CR1, CCR3, CCR5, CCR7, CCR8, CCR10	↑: IL-6	–	↑: VEGF, CTGF, TGF-β1, bFGF	–	↑: TIMP1, CfB, miR-31, T-bet	HLA haplotype 8.1	[10, 28, 39, 40, 42, 43]
	↓: CXCL5, CXCL7, CXCL8, CXCL9, CXCL12, CXCL13, XCL1, CCL7, CCL8, CCL16							
LC	↑: CXCL11, CXCL8, CCL3, CCL5	↑: IL-15 mRNA	–	–	↓: CD4+T, CD4+CD8+T, CD4-CD8-T;	↑: T-bet/GATA-3.	–	[10, 42]
					↑: CD8+T, CD4+γδ +T	↓: TcRβ V-J: eveness, diversity		

↑: increased indicator level; ↓: decreased indicator level; –, not mentioned; CC, collagenous colitis; LC, lymphocytic colitis; MC, microscopic colitis; CXCL, C-X-C motif chemokine ligand; CCL, chemokine c-c motif ligand; CX3CL, C-X-3-C motif chemokine ligand; CXCR, C-X-C motif chemokine receptor; TNF, tumor necrosis factor; IFN, interferon; IL, interleukin; COX, cyclooxygenases; Th, helper T-cell; CD, Cluster of Differentiation; FoxP3, forkhead box protein P3; Treg, regulatory T-cell; TREC, T-cell receptor excision circle; CX3CR, C-X-3-C motif chemokine receptor; CCR, C-C chemokine receptor; XCL, X-C motif chemokine ligand; VEGF, vascular endothelial growth factor; CTGF, connective tissue growth factor; TGF, transforming growth factor; bFGF, basic fibroblast growth factor; TIMP, tissue inhibitor of metalloproteinase; CfB, complement factor B; miR, miRNA; HLA, human leukocyte antigen; T-bet, T-box transcription factor; GATA, a class of transcriptional regulators that normally recognize the consensus sequence WGATAR (W = T or A; R = G or A); TcRβ V-J, T-cell antigen receptor beta chain variable-J.

## The interrelationship between MC and IBD

### New onset of IBD after MC diagnosis

#### Status summary of the MC-to-IBD transition

When a diagnosis of either LC or CC is established, a small proportion of patients may subsequently develop IBD, irrespective of whether it presents as Crohn's disease (CD) or ulcerative colitis (UC). However, the overwhelming majority of these reports are case reports or case series, with three of them reporting the transformations of CC to UC [44–46], four reporting the transformations of CC to CD [47–50], and one reporting the transformation of LC to UC [49]. Apart from these scattered cases, only a single recent cohort study [4] and a recent case–control study [51] have examined this conversion relationship. The cohort study was conducted as a nationwide prospective study in which researchers included 13,957 patients with MC and found a remarkable association of MC with IBD, with an adjusted hazard ratio of 12.6 (95% CI, 8.8–18.1) for CD, 17.3 (95% CI, 13.7–21.8) for UC, and 16.8 (95% CI, 13.9–20.3) for IBD, and when comparing patients with MC with their non-affected siblings, they were still at significant risk of developing IBD. Another nationwide case–control study that included 15,597 patients with MC explored the relationship between 16 autoimmune diseases and MC, and found a significant association with MC and UC as well as CD, along with a higher risk of UC. Another study examining the link between MC and IBD based on clinical and pathological features revealed that the interval required for progression from CC or LC to IBD was relatively short, with an average of 14 months [52]. In this direction of disease evolution, the mean age of new-onset IBD patients is 66.5 years, which is inconsistent with the usual age of onset of IBD. These findings may suggest that MC may be the initial manifestation in a proportion of older patients with IBD, as an attenuated form of IBD undergoing transition and transformation.

#### Possible mechanisms involved in the MC-to-IBD conversion

##### Genetic overlap

In the genome-wide association study (GWAS) carried out in UK Biobank [53], the researchers used single nucleotide polymorphisms (SNPs) to calculate genetic risk scores (GRS) for IBD and its two phenotypes, CD and UC, to investigate the possible genetically related overlap between MC and IBD, and to compare their mean GRS to MC and controls without any related disease, respectively, comparisons. The results presented using the OR and 95% CI found genetic overlap between MC and both CD (*P* = 0.035) and IBD (*P* = 0.019) but failed to find a statistically significant higher genetic risk in UC (*P* = 0.261). A further genetic association study yielded 15 pleiotropic signals and these results identified a common genetic link between CC and IBD and celiac disease [54]. Similarly, another systematic gene discovery study also documented the genetic overlap between CC and IBD and its two subtypes, and suggested that there may be shared genetic risk loci between CC and IBD, rather than resemblance in the human leukocyte antigen (HLA) region [55]. The above genetic correlation studies provide a scientific basis for possible mechanisms of interaction between MC and IBD at the level of genetic susceptibility (Table 3).

**Table 3.** Association of MC (both CC and LC) with genetic aspects of IBD (both CD and UC) and celiac disease

Genetically linked diseases	Author, year	Country	Study design	Study cohort	Detection methods	Genetic linkage to MC (both CC and LC)	*P*-value
MC and IBD	Green *et al.* [53], 2019	UK	GWAS	483 MC cases and 450,616 controls	Genetic risk scores calculated using ORs for previously published SNPs	Mean (95% CI) score of 0.9286 (0.9237–0.9335) for patients with MC; 0.9230 (0.9229–0.9231) for controls	0.019
MC and CD						Mean (95% CI) score of 0.9688 (0.9632–0.9739) for patients with MC; 0.9634 (0.9632–0.9636) for controls	0.035
CC, CD, and UC	Stahl *et al.* [54], 2021	US	Genetic association study	84,922 SNPs for 804 CC cases, 11,700 celiac disease cases, 17,342 CD cases, 13,436 UC cases, and 27,101 controls	Using ASSET and CPBayes to identify loci with common genetic effects	rs6702421 chr1:195651049 near *CRB1*, *DENND1B*; rs1981525 chr5:131699561 near SLC22A4/5, and rs10114531 chr9:4988855 near *INSL4/6*, *TAK2*	NA
CC, celiac disease, and CD						rs10806425 chr6:90868580 near *BACH2*, rs9482850 chr6:128307943 near *PTPRK*, and rs1250566 chr10:80702538 near *ZMIZ1*	
CC, celiac disease, and UC						rs4142969 chr6:138003826 and rs6927172 chr6:138000928	
CC and celiac disease						rs4525910 chr3:161108490 near *IL12A*, rs653178 chr12:110492139 near *ATXN2*, and rs516246 chr19:53897984 near *RASIP1*	
CC, celiac disease, CD, and UC						rs12131796, chr1:199137377 near *KIF21B*; rs12656877, chr5:141418152 near *NDFIP1*, rs56086356 chr11:127881686 near *ETS1*, and rs243317 chr16:11254549 near *PRM1/2/3*, *SOCS1*, and *TNP2*	
CC and IBD	Westerlind *et al.* [55], 2017	Germany, Sweden	Genetic association study	314 patients with CC and 4,299 controls from three separate North European cohorts	Retrieval of summary statistics from the GWAS/Immunochip meta-analysis from the IIBDGC and comparison with the CC Immunochip summary statistics from the current study to perform the validated SECA	Significant associations of IBD/CD/UC risk loci in CC: rs2930047, rs3851228, rs6920220, rs9297145, rs6651252, rs10761659, rs1250546, and rs1893217	<0.001
CC and CD							<0.001
CC and UC							<0.01
CC and celiac disease	Fernández-Bañares *et al.* [86], 2005	Spain	Genetic association study	25 patients with LC, 34 with CC, and 70 healthy controls	HLA-DQ2 and HLA-DQ8 were investigated in patients with MC using PCR-SSP	24.3% healthy controls were DQ2-positive, 32.3% with CC were DQ2-positive	0.38
LC and celiac disease						24.3% healthy controls were DQ2-positive, 48% patients with LC were DQ2-positive	0.027
MC and celiac disease	Fine *et al.* [85], 2000	US	Genetic association study	53 patients with MC and 429 normal controls	Serological cytotoxicity methods and PCR based DNA sequence-specific primer methods to detect HLA typing	HLA-DQ2 or DQ1,3 is more common in patients with MC compared with controls	<0.02

MC, microscopic colitis; CC, collagenous colitis; LC, lymphocytic colitis; IBD, inflammatory bowel disease; CD, Crohn's disease; UC, ulcerative colitis; GWAS, genome-wide association study; OR, odds ratio; SNP, single nucleotide polymorphism; CI, confidence interval; ASSET, Association analysis of SubSETs; CPBayes, Cross-Phenotype Bayesian meta-analysis approach; NA, not available; IIBDGC, the International Inflammatory Bowel Disease Genetics Consortium; SECA, SNP effect concordance analysis; HLA, human leukocyte antigen; PCR, polymerase chain reaction; SSP, sequence-specific primer.

##### Involvement of immunological profiles

Within the immunological landscape of MC (both CC and LC), two main types of T-cells are involved. One type is the regulatory T-lymphocytes (Treg), which control the immune responsiveness of the body (also called suppressor T-lymphocytes). In the intestinal mucosa of CC and LC, both CD4+CD25+FOXP3+ Treg and non-Treg and CD4+CD25-FOXP3+ T-cells are shown to be upregulated and the latter has also been shown to be associated with both regulatory and immunosuppressive effects [39]. Treg-secreted IL-10, known for its function in triggering immunosuppression, is also increased in expression in patients with CC and LC [39]. The second group of T-cells, Th1 and Th17 lymphocytes, are also involved in the immune response as helper T-cells. Th1 secretes mainly interferon-γ (IFN-γ) and tumor necrosis factor-α (TNF-α), whereas Th-17 secretes primarily IL-21, IL-22, and IL-17-A. Both cytokines have a role in promoting inflammation and their increment reflects the clinical activity and severity of inflammation [40]. In both CC and LC, mRNA levels of cytokines increased by flow cytometry, while no elevation of Th1 or Th17 at the cellular level or protein level was found [39].

A 14-year study including 2,324 patients with MC identified 20 cases of conversion to IBD [56]. Comparing the immunological profiles of 13 “IBD transformers” with 22 MC regressions, researchers noticed that IFN-γ, TNF-α, and the specific transcription factor T-bet for Th1 were increased in the transformers. Notably, these immune-related factors were all associated with Th1. This intriguing result indicates that in some subgroups of patients with MC, there is a spread of their pre-existing inflammation. One possible explanation is that in this subgroup, the anti-inflammatory effect of IL-10 fails, thus allowing Th1 to deregulate and thereby proliferate, secreting more pro-inflammatory factors, and leading to increased inflammation with consequent disease transformation [57].

##### Disturbed intestinal microflora

Substantial alterations in the composition and function of the gut microbiota have been found in both MC and IBD, and this microbiota disruption affects the respective pathophysiological courses of both diseases and is involved in the pathogenesis underlying each [58–60].

**Figure 1.**
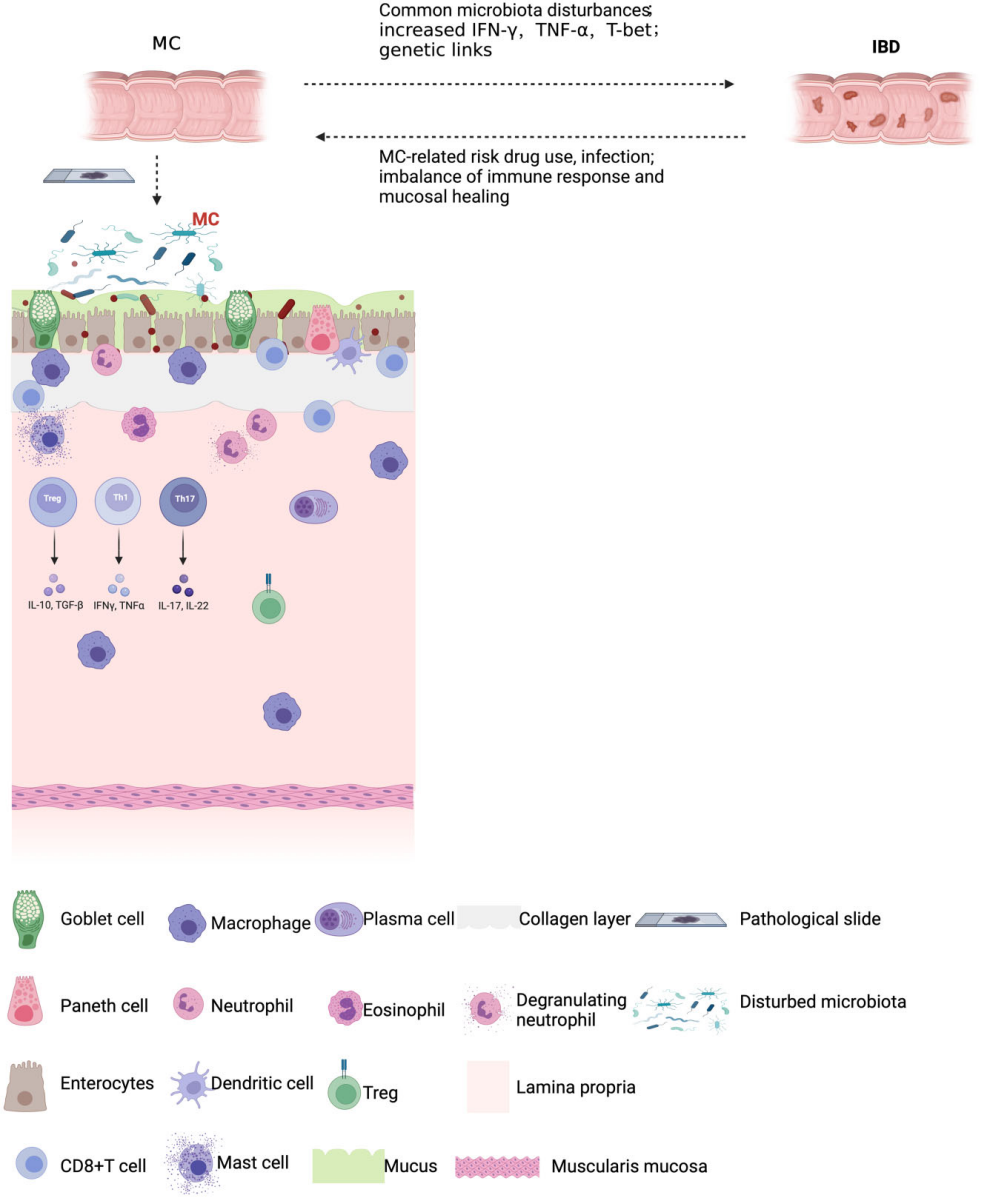
A graphical summary of the conversion of MC and IBD to each other. MC and IBD share some intestinal dysbiosis, increased pro-inflammatory factors such as IFN-γ and TNF-α in the conversion of MC to IBD, and common genetic effects between the two. The transformation of IBD to MC may then be triggered by certain risk factors such as MC-related drug use, infection, and mechanistically may be due to an imbalance of the immune response and mucosal repair. MC, microscopic colitis; IBD, inflammatory bowel disease; IFN, interferon; TNF, tumor necrosis factor; Treg, regulatory T-cell; T-bet, T-box transcription factor; IL, interleukin; TGF, transforming growth factor.

In fecal samples from patients with active MC, there was a significant decrease in biodiversity, known as alpha diversity [61]. This decrease in species richness of microorganisms was also found in patients with IBD [62] and was more prominent in patients with CD [63]. Another study comparing the microbiota of stool samples from patients with CC and IBD using Taxa-specific analysis revealed a decrease in the abundance of 10 operational taxonomic units regarding the *Ruminococcaceae* family in patients with active CC or on continuous corticosteroid treatment [64]. Nine of these species were also observed in patients with CD and four had a consistent decrease in abundance in patients with UC (Figure 1).

### New-onset MC after IBD diagnosis

#### Status summary of IBD-to-MC transition

A fair number of case reports or case series have reported that a small proportion of patients with IBD, including CD and UC, converted to CC or LC after treatment [49, 50, 65–70]. In fact, patients with IBD often experience symptoms such as diarrhea during the remission period after drug maintenance therapy, which may be confused with a relapse of IBD [65]. A retrospective observational study reported a possible shift to MC in 2.6% of patients with IBD [50]. Transformation is typically temporary and can be clinically dissipated by treating MC. In contrast to the disease transition from MC to IBD, it usually takes >10 years from the diagnosis of IBD to the detection of new-onset MC, and the average age of MC patients is usually younger [52]. This trend seems to suggest that new-onset MC patients are a continuation of IBD in the quiescent phase.

#### Risk factors and possible mechanisms

Proposed risk factors for the emergence of emerging MC may be medication use and infections [65]. In some case reports, a proportion of patients have used drugs that can be linked to the development of MC, such as NSAIDs, PPIs, and statins, and one case report even described a case of new-onset MC following high-dose chemotherapy drugs and autologous stem cell transplantation [71]. However, not all patients have a history of taking the relevant risk medication and a case series did not find an association between drug use and new-onset MC [67]. A second potential cause relates to infection, particularly *C. difficile* infection. Some patients have been reported to present with MC after refractory *C. difficile* infection [65], one of whom was treated with fecal microbial transplantation after *C. difficile* infection and eventually identified the presence of MC [69].

Little is known about the mechanisms underlying the onset of MC in patients with IBD in remission. The coexistence of some autoimmune diseases such as autoimmune thyroid disease and diabetes mellitus in some patients prior to the onset of MC seems to point to the presence of an inappropriate immune response in some affected individuals [67, 72]. During the remission period of IBD, the intestinal mucosa is in a healing state and certain risk factors such as drug use and infections may lead to an aberrant response of the intestinal mucosa to these factors, and this imbalance between the immune response and mucosal repair could be responsible for the development of MC (Figure 1).

### Management aspects in interaction with IBD

Clinicians should investigate the possibility of IBD when a patient with confirmed MC has not improved over a relatively long duration after standard treatment or has demonstrated more frequent inflammatory activity. This condition may involve very extensive lesions in some patients and therefore sufficient regard should be given to this unusual presentation [45, 67]. Furthermore, incidental detection of frequent episodes of watery diarrhea in patients with IBD in remission after decades with possible risk medications or co-infection requires vigilance for new onset of MC. Meanwhile, the suspicion of a new development should be confirmed by endoscopic observation and multiple biopsies.

## Relationship between MC and other lymphocytic disorders of the gastrointestinal tract

### Summary of the association of MC with other lymphocytic disorders of the gastrointestinal tract

About 14% of patients with MC are affected by other concurrent gastrointestinal lymphocytic disorders, including celiac disease, duodenal intraepithelial lymphocytosis (DIL), lymphocytic gastritis (LyG), and lymphocytic esophagitis (LyE) [73]. Coeliac disease is the most common concomitant autoimmune disorder in patients with MC [74] and there is an equally bidirectional relationship that can be established between the two entities. The latest case–control study revealed a >10-fold risk of celiac disease in patients with MC compared with the general population (OR = 10.15; 95% CI, 8.20–12.6) [51]. The conclusion is similar to those of other population-based and epidemiological studies [73, 75–78]. A meta-analysis summarized the prevalence of each of these two diseases in refractory cases [79]. In patients with refractory coeliac disease, the prevalence of MC was 4.5% and similarly in patients with refractory MC, the prevalence of coeliac disease was 6.7%. These results indicate an overlap of the two diseases. In patients with coeliac disease complicating MC, the individuals are usually more elderly and exhibiting significantly more duodenal mucosal atrophy [75] whereby in patients with MC presenting with concomitant coeliac disease, the patients are younger than in those without co-morbidity [73]. LyG is a histopathological pattern characterized by lymphocytosis within gastric epithelium [80]. Whereas it is not a specific disease group, it is usually seen in coincidence with other lymphocytic disorders and is closely associated with MC [73, 81]. A cross-sectional study including 3,038 patients with LyG reported that 19% of patients can develop MC as a co-morbidity [82]. DIL is the most common lymphocytic disorder of the gastrointestinal tract. Although only a small proportion of patients with DIL may co-morbidly develop MC, pathological evidence has suggested that it may manifest in ≤8% of those with MC [73].

### Possible shared etiological linkages

#### Shared genetic effects

Celiac disease is associated with genotypes such as HLA-DQ2 and DQ-8 (mainly HLA-DQ2) [83, 84]. In one study, the authors found an increase in both HLA-DQ2 and DQ-8 in celiac disease and MC compared with normal controls [85]. Another study employing polymerase chain reaction amplification using sequence-specific primers (PCR-SSP) found an increase in HLA-DQ2 in LC compared with controls, but not in CC [86]. Further confirmation of the overlap between the two diseases at the genetic level was provided by a recent study that identified 15 polygenic pleiotropic associations (using SNPs) for CC, UC, CD, and celiac disease [54]. These have demonstrated that celiac disease and MC do present a joint genetic risk. The shared immunogenic molecules allow a possible link between the two etiologically (Table 3).

#### Resembling mucosal immunological profiles

Celiac disease is a T-cell-mediated immune disorder mainly induced by gluten [87]. As a multifactorial disease, immune-related factors are central to the pathogenesis. In terms of immunopathogenesis, exposure to dietary antigens is presented to T-cells by the antigen-presenting cells (APCs) of the body, which induce intraepithelial cytotoxic CD8+ γδ, αβ T-cells to migrate to the targeted locations resulting in inflammation and injury [88, 89]. Another essential component is the involvement of helper T-cells in the lamina propria, mainly Th1, Th17, and regulatory T-cells, which differentiate and respectively produce cytokines to mediate inflammation via engagement with the APC [90–92]. Previously referred to as an increase in the immunological profile of the Th1-associated cytokine IFN-γ in patients with MC, similar alterations were demonstrated in celiac disease [93]. Also, E-cadherin was found to be significantly reduced around inflammation in celiac disease [94], which was paralleled by MC [93]. This resemblance in mucosal cytokines may explain the possible common pathophysiological mechanisms that both share in lymphocytic disorders.

### Impact on clinical management decisions

In some patients with MC coupled with coeliac disease, a gluten-free diet may be able to mitigate disease progression and decrease medication for MC [95]. Coeliac disease and MC share numerous clinical manifestations, with diarrhea being the predominant symptom. The diagnosis of coeliac disease in most patients with MC is likely to be due to a lack of response to medication or the presence of persistent diarrhea and the suspicion of other conditions, and vice versa in patients with coeliac disease [75]. Therefore, special attention needs to be drawn to possible complications in these populations. A bidirectional approach to endoscopy is also required in refractory cases when necessary to establish the diagnosis to help avoid a missed presentation. Notably, in patients with MC, intraepithelial lymphocytosis of the duodenum is occasionally observed, which may be analogous to the presentation of celiac disease [96]. Similarly in patients with celiac disease, intraepithelial lymphomatosis of the colon may also be expected in the absence of LC [97]. These highlight the relevance of random biopsy and meticulous monitoring.

## Overlap of MC and IBS

### Irritable bowel syndrome-like symptoms in patients with MC

IBS is the most common form of functional gastrointestinal disorder (FBD) with abdominal pain and altered bowel habits as the main clinical manifestations [98, 99]. It is characterized by the absence of any abnormalities on clinical examination and the current diagnostic criteria are the Rome IV criteria [100]. IBS can be divided into diarrhea-predominant IBS (IBS-D), constipation-predominant IBS (IBS-C), and IBS-mixed (IBS-M) on the basis of the number of days on which the predominant bowel habit is present in the abnormal stool pattern [101]. IBS-D presents clinically with prolonged recurrent abdominal pain and diarrhea, and similarly MC has diarrhea as the main symptom, and some patients also have abdominal pain.

There is a significant overlap regarding symptoms between the two. As the diagnosis of a patient with IBS requires the exclusion of the presence of organic gastrointestinal disease, the condition seen in MC should be referred to as IBS-like symptoms. IBS-like symptoms are not uncommon in patients with MC. Many studies have reported the prevalence of symptoms that meet the diagnostic criteria for IBS in patients with MC [102–106] whereas the prevalence varies between studies; two systematic reviews and meta-analyses have concluded that IBS-compatible symptoms are found in approximately one-third of patients with MC [14, 107]. Besides the typical abdominal pain and diarrhea, these symptoms include psychological abnormalities such as anxiety and depression [103, 108]. Compared with those without IBS-like symptoms, the quality of life of this group is more compromised and the gastrointestinal symptoms are more frequent and severe in individuals with MC [108–111]. These patients are also younger and more likely to be female [104]. Smoking may contribute to the increased risk of developing IBS-like symptoms [112].

### Detection of MC in patients with IBS

A proportion of patients with confirmed IBS are found to have pathological MC on screening colonoscopy and biopsy [113–118]. The prevalence of MC in FBD is at ∼7%, while in IBS-D, MC can be detected at 9.8% [14]. IBS-D is the type of IBS in which MC is most frequently detected, while IBS-C is rarely found [119, 120]. MC were typically detected predominantly in older women [119]. This possible overlap has also appeared to be controversial, with one meta-analysis showing that the OR of MC in patients with IBS did not reach statistical significance when compared with other patients with diarrhea [107]. However, as there are so few relevant available studies, this relationship may require more consideration. An evidence-based research has shown that the characteristics of individuals including being >50 years old, nocturnal stools, weight loss, duration of diarrhea for <12 months, and medication use are associated with increased risk of MC, and that comorbid immune disorders are also a risk factor for MC [121]. The prevalence of organic gastrointestinal disease is higher when alert symptoms are present in IBS than in its absence, but organic gastrointestinal disease may still be found in one in six patients in the population without alert features [122]. Strikingly, one-third of patients with IBS may have delayed treatment due to misdiagnosis [105] and it is estimated that 25% of patients with MC fail to receive timely biopsies in the IBS population [120].

### Management implications regarding the diagnostic overlap of MC and IBS

Management of IBS-like symptoms in MC patients is a critical issue given that one-third of MC patients develop IBS-like symptoms. Anxiety and depression as psychological disorders impair the quality of life of patients with MC and therefore the administration of psychological medications such as 5-hydroxytryptamine reuptake inhibitors may improve the prognosis of MC patients and reduce the burden of the disease. This overlap in symptoms can have a noticeable impact on the treatment of MC [103].

The detection of possible MC in patients with IBS, especially in patients with IBS-D, is paramount as it may prevent incorrect medication use and the progression of MC leading to a reduced quality of life for the patient [123]. Concerning the necessity for colonoscopy and tissue biopsy in patients with IBS because of a suspected diagnosis of MC is an issue worth noting and weighing up. On the one hand, excessive invasive testing would increase potentially unnecessary costs and lead to a psychological burden on the patient and reinforcement of the associated symptoms [14]. Hence, the diagnosis of IBS is still largely based on clinical data and simple diagnostic techniques, and colonoscopy is only performed if suspicious symptoms of organic diseases are present. On the other hand, possibly under-diagnosed MC can delay treatment because a definitive diagnosis is not carried out. As the prevalence of MC is not higher in patients with IBS than that in other diarrheal populations, it seems justified to assume that routine colonoscopy is not necessary [107]. Colonoscopy and biopsy to rule out and diagnose possible MC are indicated in patients with IBS when factors that may suggest an increased risk of MC are identified. Persistent diarrhea in older women, for example, warrants prompt colonoscopy as well as biopsy to confirm the presence of MC. The clinical background and demographic characteristics of the patient may also reduce the incidence of misdiagnosis in patients with IBS [124]. Several biomarkers including NGAL/LCN2 [125] and fecal calprotectin [126] may also play a role in the differential diagnosis of IBS and MC. However, the discriminatory value of these markers is questionable and fecal calprotectin was not associated with IBS-like symptoms in a cross-sectional study conducted by Pagoldh *et al.* [102]. Finally, both IBS and MC patients can be triggered by infection. Post-infectious IBS can occur in >10% of patients following infectious enterocolitis [127] and gastrointestinal infections are also a possible causative factor for MC. Therefore, MC detected in patients with IBS or IBS-like symptoms presented in patients with MC can be triggered by infection and this possibility needs to be clarified in clinical practice.

## Association of MC and colorectal neoplasia

### Possible reduced risk of colorectal cancer and precancerous conditions

The relationship between MC as a chronic inflammatory disease of the intestine and colorectal cancer (CRC) and precancerous lesions has been investigated (Table 4). Other intestinal chronic inflammatory diseases such as IBD [128–130] and celiac disease [131, 132] have been shown to increase the risk of CRC, although the risk of CRC in coeliac disease appears to be disputed [133, 134]. Paradoxically, the prevalence of CRC and its precancerous lesions may not only not increase in patients with MC [106, 135–137] but has been demonstrated in a notable number of studies to reduce the risk of CRC [138–144], which is a noteworthy protective effect. Cancer-related deaths were also lower in patients with MC than in matched controls [145].

**Table 4.** Studies on the association of MC (including CC and LC) with colorectal neoplastic lesions

Type of MC	Author, year	Country	Study design	Patient cohort	Age, mean ± SD, years	Colorectal neoplastic lesion type	Effect size (95% CI)	Adjustment factors
MC	Borsotti *et al.* [138], 2021	Italy	Prospective cohort study	43 (28 CC; 15 LC)	60 ± 16	Colorectal neoplasia	OR: 0.39 (0.22–0.67)	Age, gender
MC	Weimers *et al.* [139], 2021	Denmark	Nationwide cohort study	14,302 (8,437 CC; 5,865 LC)	65 ± 14	CRC	RR: 0.47 (0.38–0.59)	Charlson co-morbidity index
MC	Bergman *et al.* [140], 2020	Sweden	Population-based cohort study	11,758 (3,734 CC; 8,024 LC)	59 ± 17	CRC	HR: 0.52 (0.40–0.66)	Age, sex, county of residence, calendar year, education level, diabetes, coeliac disease
MC	Levy *et al.* [135], 2019	US	Retrospective cohort study	221 (112 CC; 109 LC)	65.7 ± 15.4	Colorectal neoplasia	OR: 1.07 (0.69–1.66) for tubular adenoma; 1.26 (0.17–9.42) for villous adenoma^a^	Age, gender, smoking, BMI
MC	Sonnenberg *et al.* [144], 2015	US	Case–control study	11,176^b^	64.2 ± 13.8	Colon polyps	OR: 0.46 (0.43–0.49) for hyperplastic polyps; 0.24 (0.19–0.30) for serrated adenomas; 0.35 (0.33–0.38) for tubular adenomas	Age, sex
MC	Tontini *et al.* [143], 2014	Italy	Prospective cohort study	43 (30 CC; 13 LC)	67 ± 15	Colorectal neoplasia	OR: 0.22 (0.05–0.97)	NA
MC	Yen *et al.* [142], 2012	US	Case–control study	647 (281 CC; 386 LC)	68.0 ± 14.7	CRC and adenoma	OR: 0.34 (0.16–0.73) for CRC; 0.52 (0.39–0.69) for colorectal adenoma	Family history of CRC, tobacco use, alcohol use, BMI
MC	Kao *et al.* [106], 2009	US	Retrospective analysis	547 (171 CC; 376 LC)	61.7^c^	CRC	NA^d^	NA
CC	Chan *et al.* [137], 1999	US	NA^e^	117	61 ± 13	CRC	NA^d^	Age, race, sex, calendar time
CC	Larsson *et al.* [141], 2019	UK; Sweden	Two-stage observational study	738; 1141	68 (58–77); 67 (57–76)^f^	Colon cancer	SIR: 0.23	Year of onset, sex, age group
CC	Bonderup *et al.* [136], 1999	Denmark	Retrospective follow-up^g^	24	NA	CRC	NA^d^	NA

MC, microscopic colitis; CC, collagenous colitis; LC, lymphocytic colitis; SD, standard deviation; CI, confidence interval; OR, odds ratio; CRC, colorectal cancer; SD, standard deviation; RR, relative risk; HR, hazard ratio; BMI, body mass index; NA, not available; SIR, standardized incidence ratio.

a Compared with the general US population, MC was not associated with an increased risk of CRC in either men (SIR 1.59, 95% CI 0.27–5.24) or women (SIR 1.97, 95% CI 0.86–3.89).

b No numbers reported for CC and LC respectively.

c No SD reported.

d All these studies did not report cases of CRC.

e A study reporting on the incidence of cancer in CC.

f The interquartile range (IQR) is reported instead of SD.

g A retrospective follow-up of patients with CC diagnosed during 1979–1990.

### Possible factors and mechanisms explaining the protective effect

First, as the diagnosis of MC requires colonoscopy, the increased activity in the utilization of colonoscopy may result in an improved detection rate of early lesions in CRC, which is a protective effect from colonoscopy [146]. Second, medications such as NSAIDs and statins that may be prescribed in patients with MC have a preventive effect on the development of CRC [147, 148]. In addition, body mass index (BMI) is relatively low in patients with MC and high BMI is positively associated with the development of CRC, thus lowering the risk of CRC [139]. Furthermore, the increased intestinal intraepithelial lymphocytes of the MC enhance immune surveillance and thus reduce the prevalence of CRC. This form of immune surveillance is primarily engaged by T-lymphocytes in the epithelium; when carcinogenic antigens are present in the gut, it first activates the NKG2d receptor on natural killer cells, which in turn can activate γδ T-cells to kill cells in the presence of DNA damage and stress [142]. Such T-cells are essential for the clearance of mutant and abnormal cells [138]. The absence of γδ T-cells leaves the mice model vulnerable to epithelial tumors, suggesting a protective role for the T-cells in immune surveillance [149]. Finally, a comparable process of carcinogenesis may not have been present in MC as in IBD. NF-κB, a transcription factor for inducible nitric oxide synthase (iNOS), may mediate the mucosal damage caused by colonic inflammation. Over-activation is found in both MC and IBD, whereas in MC it is confined to the intestinal epithelium and in UC it spreads to macrophages in the lamina propria [150]. This indicates that chronic inflammation in MC is not as severe as in IBD and may not lead to epithelial damage and dysfunction [138]. Chronic inflammation can be responsible for cancer development through DNA damage but the severity of inflammation in MC, which is not as significant as in IBD, probably does not activate the relevant carcinogenic pathways [135, 151].

## Conclusions

As a relatively recently understood immune-mediated chronic inflammatory disease of the large intestine, MC is associated with several gastrointestinal disorders. MC may interact with classical IBD as an attenuated form of IBD and the two may be convertible. Genetic, immunological, and gut microbiological factors may be involved in this process. Both MC and celiac disease, as lymphocytic disorders, can occur in conjunction with each other. Clinical misdiagnosis is possible between MC and IBS due to the similarity of symptoms and diagnostic overlap. MC is likely to be detected in patients with IBS and should be ruled out by colonoscopy and biopsy when suspicious symptoms point to MC, and IBS-like symptoms in patients with MC should be addressed to improve their quality of life. Patients with MC have a reduced risk of CRC and colonic adenoma, and screening for CRC in individuals with MC is therefore not required. Gastrointestinal infections and increased risk of MC are associated. *Clostridiumdifficile* and *C. concisus* infections significantly increase the risk of MC, whereas *H. pylori* demonstrates an inverse relationship.

## Authors’ Contributions

Y.L. proposed the idea for the article, carried out the literature search, and wrote the manuscript, as well as prepared the illustrations and tables. M.C. revised the manuscript as the corresponding author and provided comments. All authors read and approved the final version of the manuscript.

## Funding

None.
